# The genome sequence of the devil’s coach horse, *Ocypus olens* (Müller, 1764)

**DOI:** 10.12688/wellcomeopenres.17342.1

**Published:** 2021-11-03

**Authors:** Liam Crowley, University of Oxford and Wytham Woods Genome Acquisition Lab, Darwin Tree of Life Barcoding collective, Wellcome Sanger Institute Tree of Life programme, Wellcome Sanger Institute Scientific Operations: DNA Pipelines collective, Tree of Life Core Informatics collective, Darwin Tree of Life Consortium

**Affiliations:** 1 Department of Zoology, University of Oxford, Oxford, UK

**Keywords:** Ocypus olens, devil’s coach horse, genome sequence, chromosomal

## Abstract

We present a genome assembly from an individual female *Ocypus olens *(the devil’s coach horse; Arthropoda; Insecta; Coleoptera; Staphylinidae). The genome sequence is 1,084 megabases in span. The majority (98.81%) of the assembly is scaffolded into 20 chromosomal pseudomolecules, with the X sex chromosome assembled.

## Species taxonomy

Eukaryota; Metazoa; Ecdysozoa; Arthropoda; Hexapoda; Insecta; Pterygota; Neoptera; Endopterygota; Coleoptera; Polyphaga; Staphyliniformia; Staphylinidae; Staphylininae group; Staphylininae; Staphylinini; Ocypus; *Ocypus olens* (Müller, 1764) (NCBI:txid662956).

## Background

The devil's coach horse, *Ocypus olens*, is a large, all-black rove beetle. Reaching up to 32 mm, it is the largest beetle in the family Staphylinidae in the UK, and one of the largest worldwide. It’s widespread and generally common across the Palaeartic and North Africa, including throughout mainland UK. It has been introduced to North America and Australasia. It can be found across a range of different habitats, especially damp woodland, grassland, brownfield sites and gardens. The devil’s coach horse is largely nocturnal, sheltering under leaf litter, logs and stones during the day. It is a generalist predator as both a larva and adult, feeding on a wide range of invertebrate species and carrion ( Bonacci *et al.*, 2006). Adults can be found all year and overwintering occurs in this stage. Mating occurs in late summer/autumn and eggs are laid 2 to 3 weeks later ( Nield, 1976). Adults can be relatively long-lived, living up to 2 years in this stage ( Nield, 1976). When agitated, the abdomen is reared and the mandibles opened in a threat-posture. The devil’s coach horse is capable of inflicting a painful bite to humans and readily produces defensive secretions from the mouth and tip of the abdomen. This species has been associated with evil and the devil in folklore since the Middle Ages.

## Genome sequence report

**Figure 1.**
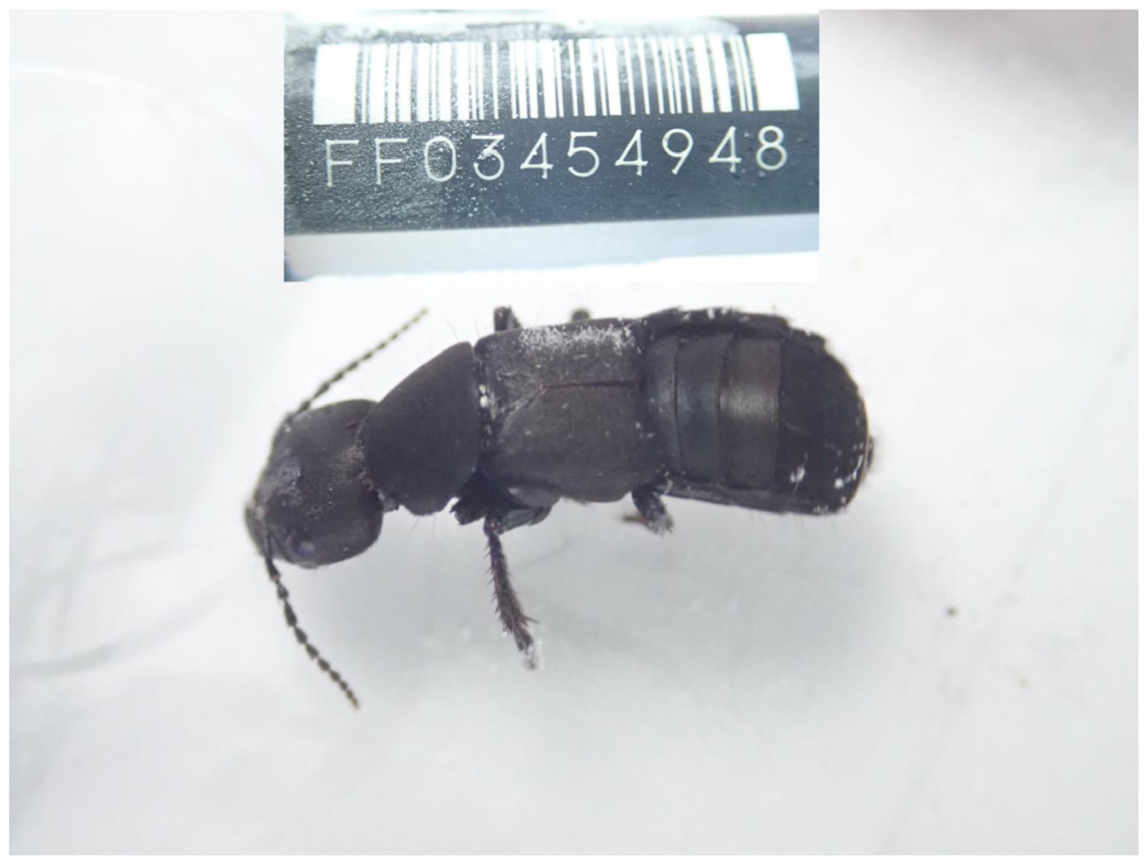
Image of the icOcyOlen1 specimen taken prior to preservation and processing.

The genome was sequenced from one female *O. olens* ( Figure 1) collected from Wytham Woods, Oxfordshire (biological vice-county: Berkshire), UK (latitude 51.775, longitude -1.326). A total of 40-fold coverage in Pacific Biosciences single-molecule long reads and 41-fold coverage in 10X Genomics read clouds were generated. Primary assembly contigs were scaffolded with chromosome conformation Hi-C data. Manual assembly curation corrected 541 missing/misjoins and removed 28 haplotypic duplications, reducing the assembly length by 0.45% and the scaffold number by 64.24%, and increasing the scaffold N50 by 188.60%.

**Figure 2.**
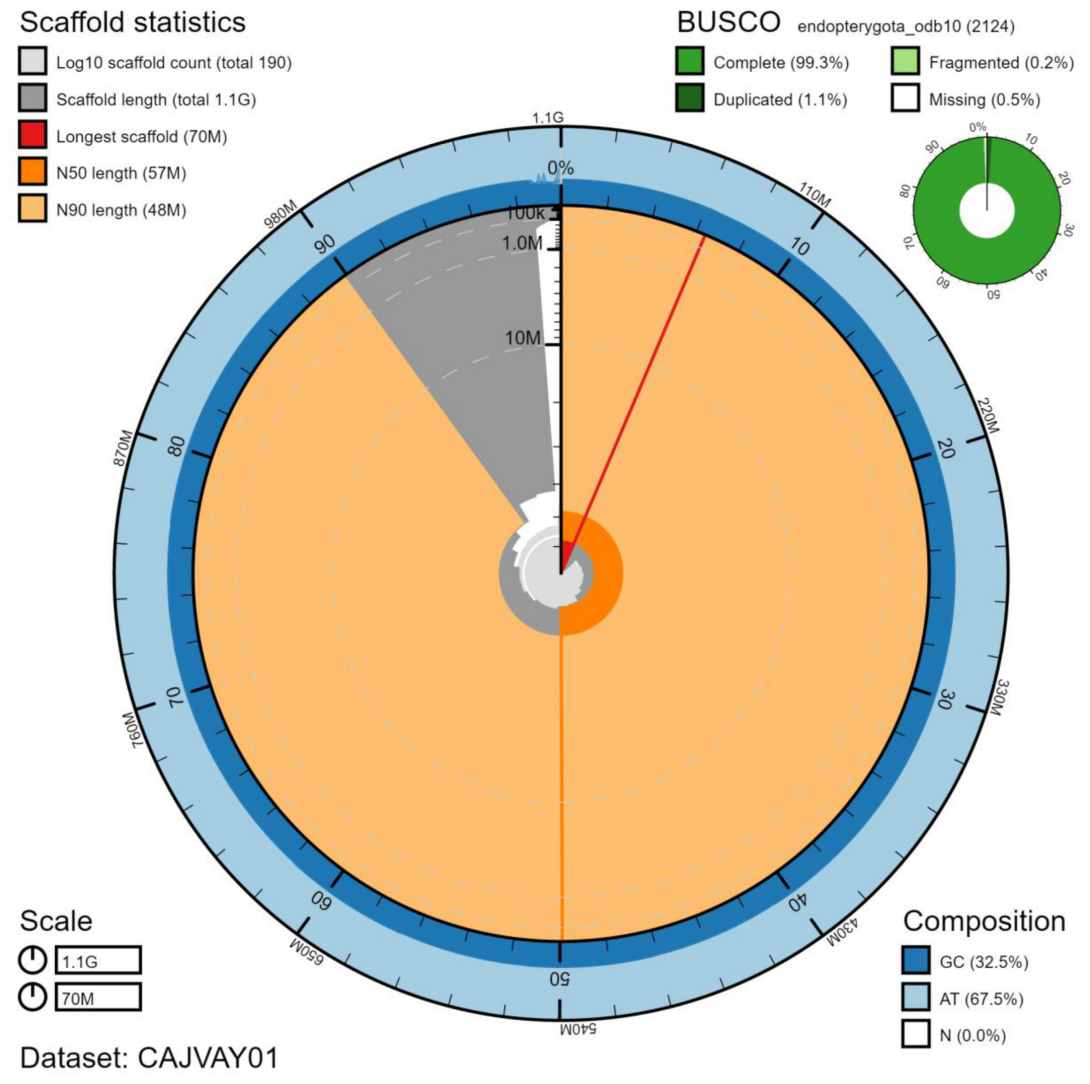
Genome assembly of *Ocypus olens*, icOcyOlen1.1: metrics. The BlobToolKit Snailplot shows N50 metrics and BUSCO gene completeness. The main plot is divided into 1,000 size-ordered bins around the circumference with each bin representing 0.1% of the 1,083,870,412 bp assembly. The distribution of scaffold lengths is shown in dark grey with the plot radius scaled to the longest scaffold present in the assembly (69,741,075 bp, shown in red). Orange and pale-orange arcs show the N50 and N90 scaffold lengths (57,303,393 and 48,121,331 bp), respectively. The pale grey spiral shows the cumulative scaffold count on a log scale with white scale lines showing successive orders of magnitude. The blue and pale-blue area around the outside of the plot shows the distribution of GC, AT and N percentages in the same bins as the inner plot. A summary of complete, fragmented, duplicated and missing BUSCO genes in the endopterygota_odb10 set is shown in the top right.. An interactive version of this figure is available at https://blobtoolkit.genomehubs.org/view/icOcyOlen1.1/dataset/CAJVAY01/snail.

The final assembly has a total length of 1,084 Mb in 187 sequence scaffolds with a scaffold N50 of 57.3 Mb ( Table 1). The majority, 98.81%, of the assembly sequence was assigned to 20 chromosomal-level scaffolds, representing 9 autosomes (numbered by sequence length), and the X sex chromosome ( Figure 2– Figure 5; Table 2). The order and orientation of scaffolds in the centromeric regions is less certain than in the rest of the assembly. The assembly has a BUSCO v5.1.2 ( Manni *et al.*, 2021) completeness of 99.3% (single 98.2%, duplicated 1.1%) using the endopterygota_odb10 reference set. While not fully phased, the assembly deposited is of one haplotype. Contigs corresponding to the second haplotype have also been deposited.

**Table 1.** Genome data for *Ocypus olens*, icOcyOlen1.1.

*Project accession data*	
Assembly identifier	icOlcyOlen.1
Species	*Ocypus olens*
Specimen	icOcyOlen1
NCBI taxonomy ID	NCBI:txid662956
BioProject	PRJEB45196
BioSample ID	SAMEA7520211
Isolate information	Female, whole organism
*Raw data accessions*	
PacificBiosciences SEQUEL II	ERR6412375, ERR6590589
10X Genomics Illumina	ERR6054955-ERR6054958
Hi-C Illumina	ERR6054776
Illumina polyA RNA-Seq	ERR6286735
*Genome assembly*	
Assembly accession	GCA_910593695.1
Accession of alternate haplotype	GCA_910593855.1
Span (Mb)	1,084
Number of contigs	733
Contig N50 length (Mb)	4.6
Number of scaffolds	187
Scaffold N50 length (Mb)	57.3
Longest scaffold (Mb)	69.7
BUSCO * genome score	C:99.3%[S:98.2%,D:1.1%],F: 0.2%,M:0.5%,n:2124

*BUSCO scores based on the endopterygota_odb10 BUSCO set using v5.1.2. C= complete [S= single copy, D=duplicated], F=fragmented, M=missing, n=number of orthologues in comparison. A full set of BUSCO scores is available at https://blobtoolkit.genomehubs.org/view/icOcyOlen1.1/dataset/CAJVAY01/busco.

**Figure 3.**
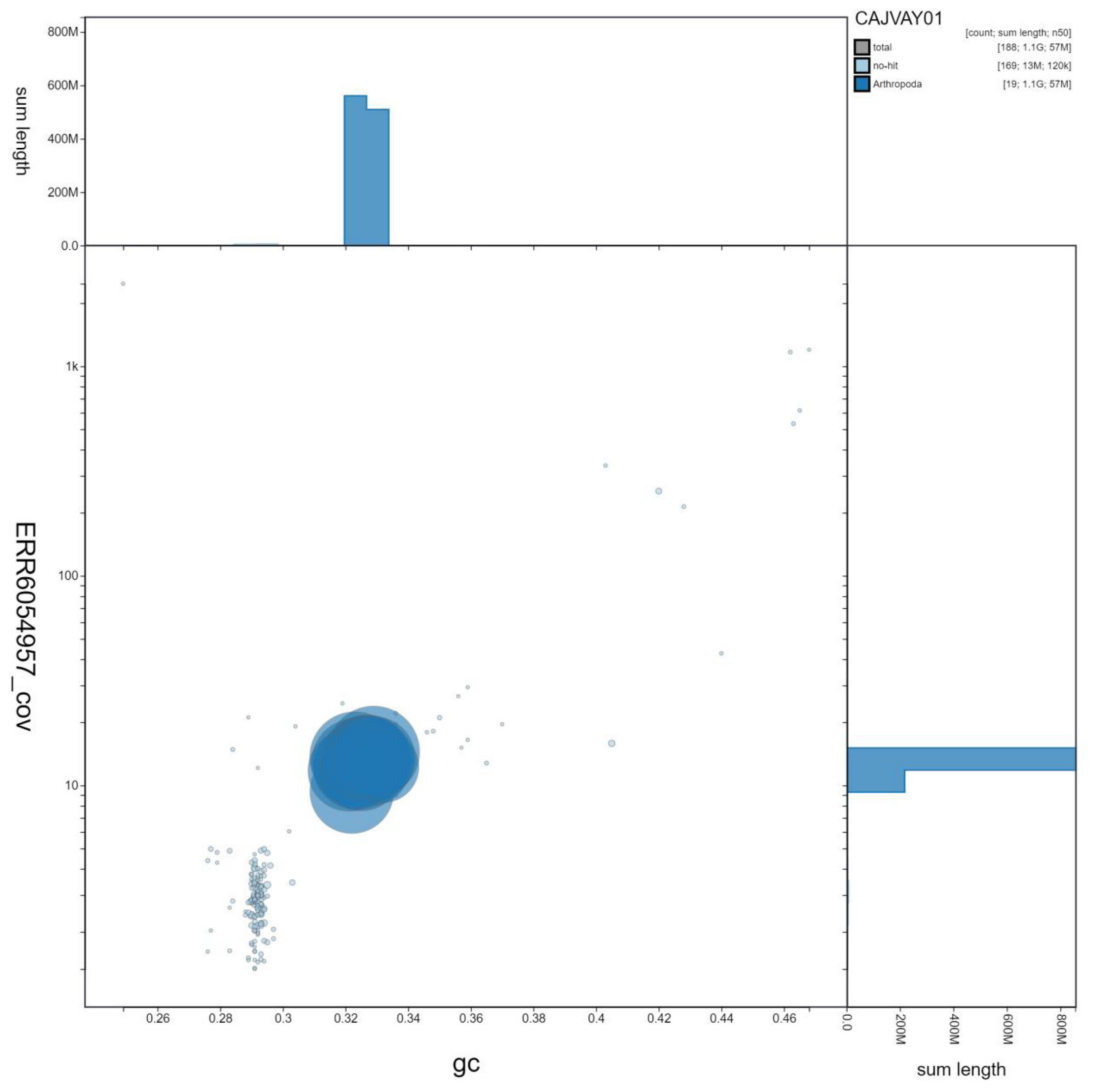
Genome assembly of *Ocypus olens*, icOcyOlen1.1: GC coverage. BlobToolKit GC-coverage plot. Scaffolds are coloured by phylum. Circles are sized in proportion to scaffold length Histograms show the distribution of scaffold length sum along each axis. An interactive version of this figure is available at https://blobtoolkit.genomehubs.org/view/icOcyOlen1.1/dataset/CAJVAY01/blob.

**Figure 4.**
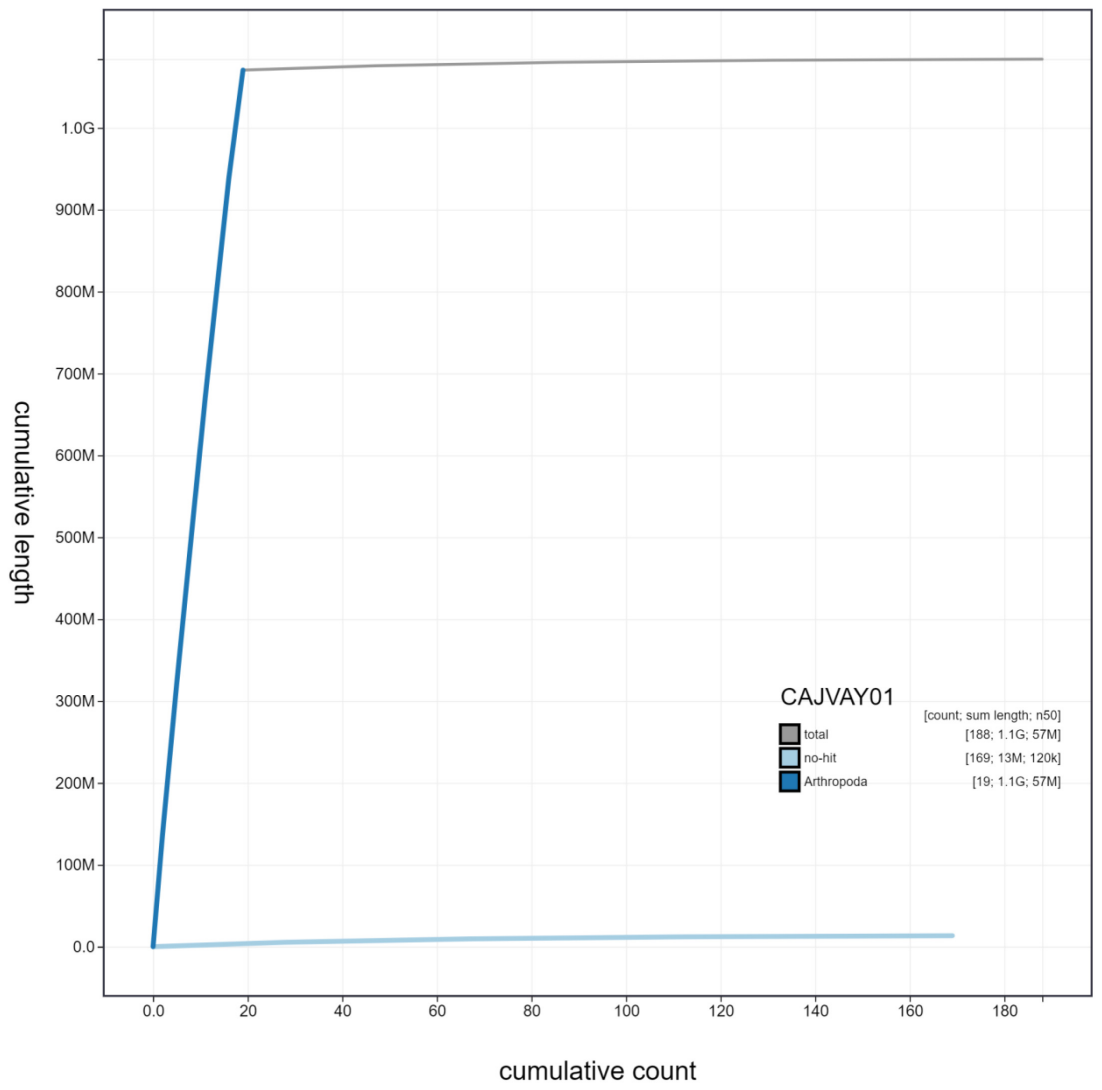
Genome assembly of *Ocypus olens*, icOcyOlen1.1: cumulative sequence. BlobToolKit cumulative sequence plot. The grey line shows cumulative length for all scaffolds. Coloured lines show cumulative lengths of scaffolds assigned to each phylum using the buscogenes taxrule. An interactive version of this figure is available at https://blobtoolkit.genomehubs.org/view/icOcyOlen1.1/dataset/CAJVAY01/cumulative.

**Figure 5.**
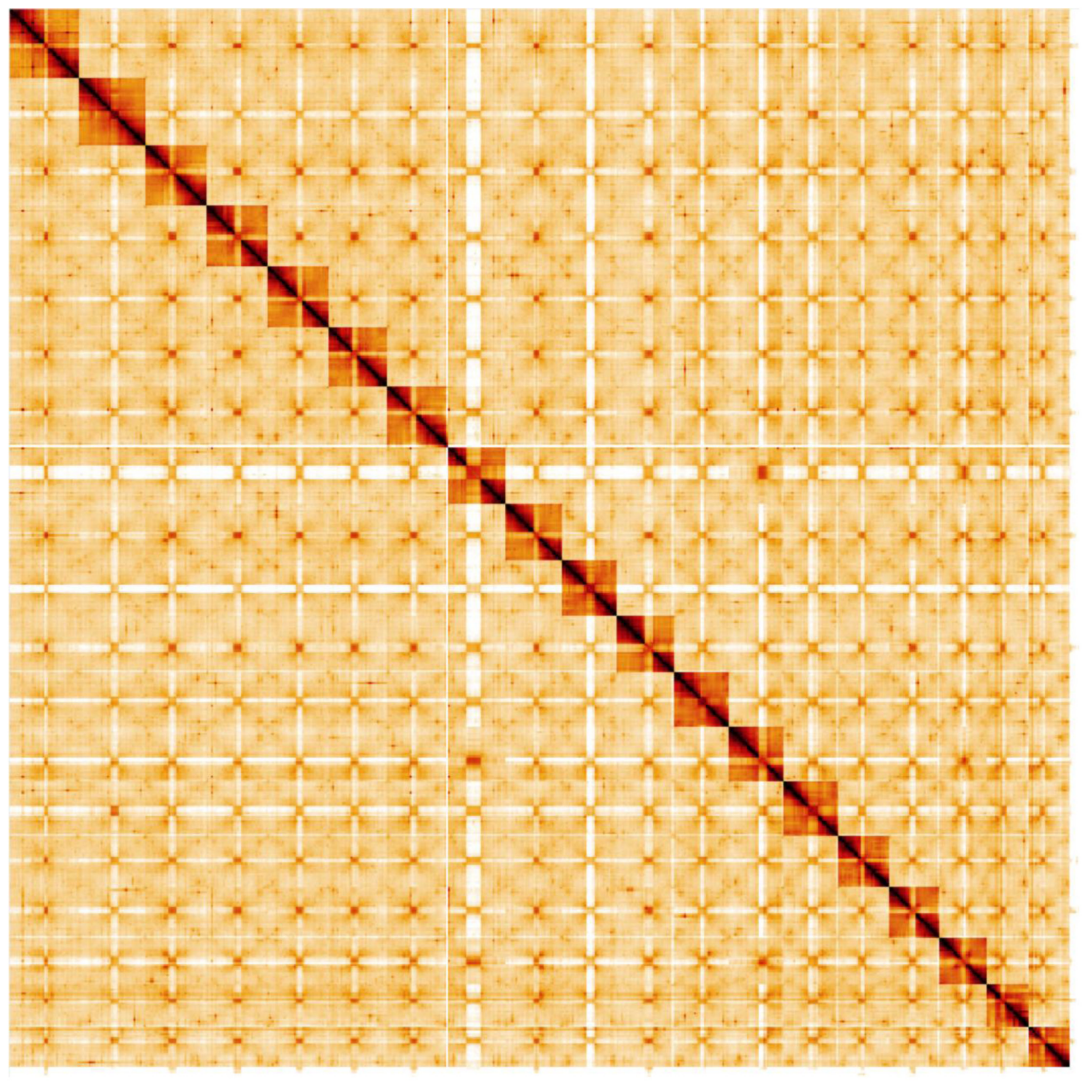
Genome assembly of *Ocypus olens*, icOcyOlen1.1: Hi-C contact map. Hi-C contact map of the icOcyOlen.1 assembly, visualised in HiGlass.

**Table 2.** Chromosomal pseudomolecules in the genome assembly of *Ocypus olens*, icOcyOlen1.1.

INSDC accession	Chromosome	Size (Mb)	GC%
OU343047.1	1	69.74	32.9
OU343048.1	2	67.56	32.3
OU343049.1	3	62.12	32.6
OU343050.1	4	61.53	32.3
OU343051.1	5	61.09	32.7
OU343052.1	6	60.89	32.7
OU343053.1	7	59.74	32.7
OU343054.1	8	57.84	32.2
OU343055.1	9	57.30	32.7
OU343056.1	10	56.54	32.3
OU343058.1	12	55.64	32.3
OU343059.1	13	54.63	32.5
OU343060.1	14	54.47	32.1
OU343061.1	15	52.48	32.9
OU343062.1	16	50.26	32.8
OU343063.1	17	48.12	32.4
OU343064.1	18	42.33	32.4
OU343065.1	19	41.70	33.2
OU343057.1	X	56.49	32.9
OU343066.1	MT	0.02	25.2
-	Unplaced	13.37	29.9

## Methods

### Sample acquisition and nucleic acid extraction

A single female *O. olens* was collected from Wytham Woods, Oxfordshire (biological vice-county: Berkshire), UK (latitude 51.775, longitude -1.326) by Liam Crowley, University of Oxford, using a pooter. The sample was identified by the same individual, snap-frozen on dry ice and stored using a CoolRack.

DNA was extracted from the whole organism of icOcyOlen1 at the Wellcome Sanger Institute (WSI) Scientific Operations core from the whole organism using the Qiagen MagAttract HMW DNA kit, according to the manufacturer’s instructions. Following this, further DNA was extracted for a PacBio top-up. Tissue was cryogenically disrupted to a fine powder using a Covaris cryoPREP Automated Dry Pulveriser, receiving multiple impacts. Fragment size analysis of 0.01–0.5 ng of DNA was then performed using an Agilent FemtoPulse. High molecular weight (HMW) DNA was again extracted using the Qiagen MagAttract HMW DNA extraction kit. HMW DNA was sheared into an average fragment size between 12–20 kb in a Megaruptor 3 system with speed setting 30. Sheared DNA was purified by solid-phase reversible immobilisation using AMPure PB beads with a 1.8X ratio of beads to sample to remove the shorter fragments and concentrate the DNA sample. The concentration of the sheared and purified DNA was assessed using a Nanodrop spectrophotometer and Qubit Fluorometer and Qubit dsDNA High Sensitivity Assay kit. Fragment size distribution was evaluated by running the sample on the FemtoPulse system.

RNA was extracted from the whole organism in the Tree of Life Laboratory at the WSI using TRIzol (Invitrogen), according to the manufacturer’s instructions. RNA was then eluted in 50 μl RNAse-free water and its concentration assessed using a Nanodrop spectrophotometer and Qubit Fluorometer using the Qubit RNA Broad-Range (BR) Assay kit. Analysis of the integrity of the RNA was done using Agilent RNA 6000 Pico Kit and Eukaryotic Total RNA assay.

### Sequencing

Pacific Biosciences HiFi circular consensus and 10X Genomics Chromium read cloud sequencing libraries were constructed according to the manufacturers’ instructions. Poly(A) RNA-Seq libraries were constructed using the NEB Ultra II RNA Library Prep kit. Sequencing was performed by the Scientific Operations core at the Wellcome Sanger Institute on Pacific Biosciences SEQUEL II (HiFi), Illumina HiSeq X (10X) and Illumina HiSeq 4000 (RNA-Seq) instruments. Hi-C data were generated from head tissue using the Arima v2 Hi-C kit and sequenced on HiSeq X.

### Genome assembly

Assembly was carried out with Hifiasm ( Cheng *et al.*, 2021); haplotypic duplication was identified and removed with purge_dups ( Guan *et al.*, 2020). One round of polishing was performed by aligning 10X Genomics read data to the assembly with longranger align, calling variants with freebayes ( Garrison & Marth, 2012). The assembly was then scaffolded with Hi-C data ( Rao *et al.*, 2014) using SALSA2 ( Ghurye *et al.*, 2019). The assembly was checked for contamination and corrected using the gEVAL system ( Chow *et al.*, 2016) as described previously ( Howe *et al.*, 2021). Manual curation ( Howe *et al.*, 2021) was performed using gEVAL, HiGlass ( Kerpedjiev *et al.*, 2018) and Pretext. The mitochondrial genome was assembled using MitoHiFi ( Uliano-Silva *et al.*, 2021). The genome was analysed and BUSCO scores generated within the BlobToolKit environment ( Challis *et al.*, 2020). Table 3 contains a list of all software tool versions used, where appropriate.

**Table 3.** Software tools used.

Software tool	Version	Source
Hifiasm	0.12	Cheng *et al.*, 2021
purge_dups	1.2.3	Guan *et al.*, 2020
SALSA2	2.2	Ghurye *et al.*, 2019
longranger align	2.2.2	https://support.10xgenomics.com/genome-exome/ software/pipelines/latest/advanced/other-pipelines
freebayes	1.3.1-17-gaa2ace8	Garrison & Marth, 2012
MitoHiFi	1.0	Uliano-Silva *et al.*, 2021
gEVAL	N/A	Chow *et al.*, 2016
HiGlass	1.11.6	Kerpedjiev *et al.*, 2018
PretextView	0.1.x	https://github.com/wtsi-hpag/PretextView
BlobToolKit	2.6.2	Challis *et al.*, 2020

### Ethics/compliance issues

The materials that have contributed to this genome note have been supplied by a Darwin Tree of Life Partner. The submission of materials by a Darwin Tree of Life Partner is subject to the Darwin Tree of Life Project Sampling Code of Practice. By agreeing with and signing up to the Sampling Code of Practice, the Darwin Tree of Life Partner agrees they will meet the legal and ethical requirements and standards set out within this document in respect of all samples acquired for, and supplied to, the Darwin Tree of Life Project. Each transfer of samples is further undertaken according to a Research Collaboration Agreement or Material Transfer Agreement entered into by the Darwin Tree of Life Partner, Genome Research Limited (operating as the Wellcome Sanger Institute), and in some circumstances other Darwin Tree of Life collaborators.

## Data availability

European Nucleotide Archive: Ocypus olens (Devil's coach horse). Accession number PRJEB45196; https://identifiers.org/ena.embl/PRJEB45196.

The genome sequence is released openly for reuse. The *O. olens* genome sequencing initiative is part of the Darwin Tree of Life (DToL) project. All raw sequence data and the assembly have been deposited in INSDC databases. The genome will be annotated using the RNA-Seq data and presented through the Ensembl pipeline at the European Bioinformatics Institute. Raw data and assembly accession identifiers are reported in Table 1.

## Author information

Members of the University of Oxford and Wytham Woods Genome Acquisition Lab are listed here: https://doi.org/10.5281/zenodo.4789929.

Members of the Darwin Tree of Life Barcoding collective are listed here: https://doi.org/10.5281/zenodo.4893704.

Members of the Wellcome Sanger Institute Tree of Life programme collective are listed here: https://doi.org/10.5281/zenodo.5377053.

Members of Wellcome Sanger Institute Scientific Operations: DNA Pipelines collective are listed here: https://doi.org/10.5281/zenodo.4790456.

Members of the Tree of Life Core Informatics collective are listed here: https://doi.org/10.5281/zenodo.5013542.

Members of the Darwin Tree of Life Consortium are listed here: https://doi.org/10.5281/zenodo.4783559.
