# Supplementary material for: The genome sequence of the devil’s coach horse beetle, Ocypus olens (Müller, 1764)
Source: Wellcome Open Res. 2024 Mar 21;6:293. Originally published 2021 Nov 3. [Version 2] doi: 10.12688/wellcomeopenres.17342.2 (PMC9069170; doi:10.12688/wellcomeopenres.17342.2)
Supplement: Peer review history

# Reviewer report: Reviewer response for version 1

**DOI:** 10.21956/wellcomeopenres.19172.r50199

**Published:** 2022-05-04

**Authors:** Robert Waterhouse

**Affiliations:** 1 Department of Ecology and Evolution, University of Lausanne, Lausanne, Switzerland

The genome Data Note for the devil’s coach horse rove beetle presents a clear and comprehensive description of all the steps taken to generate the chromosomal-level assembly spanning just over 1 Gbp with almost 99% assigned to chromosomes including the X chromosome. As a species of ecological interest and as member of the largest order of insects, the rationale for generating these genomic resources is clear. The described data collection and analysis methods follow the best practices in the field and have delivered a high-quality complete and accurate chromosome-level reference genome, with data made available through the European Nucleotide Archive.

 Additional points:

- “representing 9 autosomes” – should this not be 19 autosomes?
- In the Hi-C contact map, is it only showing the 19 autosomes?
- Could there be an error in the total scaffold count, because the counts start at zero and go to 187, this means there are 188 scaffolds not 187 – please check.
- “The genome will be annotated using the RNA-Seq data and presented through the Ensembl pipeline at the European Bioinformatics Institute.” – it seems that this has now been done, so this statement could be updated in the revision.
- If it were made clear that ‘icOcyOlen1’ is the sample ID then the ‘extracted from the whole organism of icOcyOlen1’ sentence would make more sense.

Are sufficient details of methods and materials provided to allow replication by others?

Yes

Is the rationale for creating the dataset(s) clearly described?

Yes

Are the datasets clearly presented in a useable and accessible format?

Yes

Are the protocols appropriate and is the work technically sound?

Yes

Reviewer Expertise:

arthropod comparative and evolutionary genomics

I confirm that I have read this submission and believe that I have an appropriate level of expertise to confirm that it is of an acceptable scientific standard.

# Reviewer report: Reviewer response for version 1

**DOI:** 10.21956/wellcomeopenres.19172.r46916

**Published:** 2021-11-10

**Authors:** Duane D McKenna, Xuankun Li

**Affiliations:** 1 Department of Biological Sciences, University of Memphis, Memphis, TN, USA; 2 University of Memphis Center for Biodiversity Research, Memphis, TN, USA

This manuscript entitled ‘The genome sequence of the devil’s coach horse, *Ocypus olens* (Müller, 1764)’ reports a new chromosomal-scale genome assembly of a rove beetle (family Staphylinidae). The latest methods for genome sequencing, assembly, annotation, and characterization were used in this study, and they are adequately documented in the paper. The sequencing results and assembly are clearly reported and interpreted, and appropriately illustrated, and the data is available as described. Only a few minor edits to the text (detailed below) are suggested before publication.

**Title: **

- There is no indication in the title that this is a beetle, or even an insect. Please consider revising the title to include the word “beetle” after the common name, and/or add “(Coleoptera: Staphylinidae)” to the title in reference to the order and family of beetle sequenced.

**Species Taxonomy: **

- Since you indicate both the author and year of publication for the name *Ocypus olens*, you should really include a citation to the original description. Alternatively, you could remove the year and only indicate the author last name.

**Background:**

- Line 4: Replace “It’s” with “It is”.
- Line 5: Delete “and North Africa”. By definition, North Africa is part of the Palaearctic.
- Line 6: A citation is needed when referencing the introduced range.

**Genome Sequence Report: **

- Lines 2-3: The collection data appears in both this section and the Methods. This redundancy seems unnecessary.

**Methods:**

- Line 1 in the second paragraph of the Methods: Delete “from the whole organism of icOcyOlen1.” This is redundant with mention of extraction “from the whole organism” later in the same sentence.
- Was RNA extracted from the same “whole organism” as DNA? Please clarify. The same issue needs clarifying where “head tissue” is mentioned in reference to Hi-C data at the bottom of page 6.
- Figure 2 legend: 3 ^rd^ to last sentence: There is an excess period at the end of the sentence.
- Figure 3 legend: A period is missing from the end of the sentence ending in “length”.

**Data availability:**

- The scientific name “Ocypus olens” should be italicized.

Are sufficient details of methods and materials provided to allow replication by others?

Yes

Is the rationale for creating the dataset(s) clearly described?

Yes

Are the datasets clearly presented in a useable and accessible format?

Yes

Are the protocols appropriate and is the work technically sound?

Yes

Reviewer Expertise:

insect systematics and evolution (primarily beetles), insect-plant interactions, gene and genome evolution, chemosensation, digestive physiology

We confirm that we have read this submission and believe that we have an appropriate level of expertise to confirm that it is of an acceptable scientific standard.
